# Polysaccharides Cell Wall Architecture of Mucorales

**DOI:** 10.3389/fmicb.2019.00469

**Published:** 2019-03-19

**Authors:** Karine Lecointe, Marjorie Cornu, Jordan Leroy, Pauline Coulon, Boualem Sendid

**Affiliations:** ^1^ Lille Inflammation Research International Center, UMR 995 Inserm, Fungal Associated Invasive and Inflammatory Diseases, CHU Lille, Lille University, Lille, France; ^2^ Laboratory of Parasitology and Mycology, Institute of Microbiology, CHU Lille, Lille, France

**Keywords:** *Mucorales*, polysaccharides, cell wall, glucuronic acid, glyco-enzymes

## Abstract

Invasive fungal infections are some of the most life-threatening infectious diseases in the hospital setting. In industrialized countries, the most common fungal species isolated from immunocompromised patients are *Candida* and *Aspergillus* spp. However, the number of infections due to *Mucorale*s spp. is constantly increasing and little is known about the virulence factors of these fungi. The fungal cell wall is an important structure protecting fungi from the environment. A better knowledge of its composition should improve our understanding of host-pathogen interactions. Cell wall molecules are involved in tissue adherence, immune escape strategies, and stimulation of host defenses including phagocytosis and mediators of humoral immunity. The fungal cell wall is also a target of choice for the development of diagnostic or therapeutic tools. The present review discusses our current knowledge on the cell wall structure of *Mucorales* in terms of the polysaccharides and glyco-enzymes involved in its biosynthesis and degradation, with an emphasis on the missing gaps in our knowledge.

## Introduction

Fungal adherence is a prerequisite for colonization and invasion of the host leading to infection. Fungi have also developed strategies to escape the host defenses. The cell wall, a dynamic structure essential for cell viability and morphogenesis, is one of the barriers that protects fungi from environmental stress (Latgé and Calderone, 2002). It contains enzymes that could be secreted to facilitate degradation of host tissues in order to release nutrients essential for fungal metabolism, fungal growth, and tissue invasion. The cell wall also contains environmental sensors allowing fungi to resist stress, osmotic pressure, or toxic molecules (Latgé, 2010). Almost 90% of the fungal cell wall is composed of polysaccharides not found in humans. These structures remain an ideal yet somewhat unrealized target for the development of new antifungal drugs. The cell wall structure of medically important opportunistic fungal pathogens has been well described (i.e., for *Aspergillus fumigatus, Candida albicans, Pneumocystis* spp., *Cryptococcus neoformans, Histoplasma capsulatum,* and *Blastomyces dermatitidis).* Fungal cell walls are organized in a similar way. Indeed, cell wall is composed of two layers: an inner layer which is the backbone part and an outer layer which is a kind of carbohydrate matrix. The backbone is composed of β-1,3- and/or 1,6-glucan and chitin bound to protein or other polysaccharides. The outer layer is more variable among the species. In yeasts such as *Candida* species, highly mannosylated glycoproteins are found on it. Concerning *Aspergillus fumigatus*, α-1,3-glucan, galactomannan, and galactosaminogalactan are found, but at conidia stage, there is a supplementary outer layer composed of hydrophobin and melanin. In *C. neoformans*, a gelatinous capsule of glucuronoxylomannan and galactoxylomannan masks polysaccharides cell wall (Gow et al., 2017). Glucan, chitin, and polymers of mannose residues are the most common polysaccharides making up the cell surface envelopes of fungal species.

However, little is known about the fine structure of the cell wall of *Mucorales* spp., a prevalent cause of fungal infection leading to significant morbidity and mortality (Walsh et al., 2004), mainly in immunocompromised and diabetic patients. The incidence of mucormycosis was underestimated in the past due to the low performance of diagnostic techniques based on conventional microbiological methods. With the recent improvement of molecular detection of fungal DNA (real-time PCR), the diagnostic is easier, non-invasive, and reliable. Mucormycosis represents the third invasive fungal infection in terms of overall mortality in France (Bitar et al., 2014). A retrospective study conducted from 1997 to 2006 provided a trend over a 10-year period at national level in France. This study showed an increasing incidence from 0.7/million in 1997 to 1.2/million in 2006 (Bitar et al., 2009). The annual incidence reported in the USA and Spain was 1.7 and 0.43/million, respectively (Petrikkos et al., 2012). The prevalence has been estimated at 0.01 to 0.2 per 100,000 inhabitants in Europe and in the USA, respectively, while this rate is 70 times higher in India (14 per 100,000 inhabitants) (Skiada et al., 2018). The most common *Mucorales* genera, such as *Rhizopus*, *Mucor,* and *Lichtheimia,* are involved in 70–80% of cases of mucormycosis, whereas the genera *Cunninghamella, Saksenaea, Rhizomucor, Apophysomyces, Syncephalastrum, Cokeromyces,* and *Actinomucor* are reported in only 1–5% of cases (Gomes et al., 2011). The main risk factors identified are hematologic malignancies (44%), trauma (15%), allografts (9%), diabetes (9%), cancers (5%), and solid organ transplantation (4%) (Skiada et al., 2011). The different clinical forms of mucormycosis are correlated with different risk factors. Sinus mucormycosis is predominant in patients with uncontrolled diabetes, while the invasive pulmonary form is more common in neutropenic patients and solid organ transplant recipients. Cerebral and disseminated forms are rare but are associated with high mortality rates (Roden et al., 2005). Cutaneous forms are mainly found in immunocompetent patients after trauma and contamination by environmental *Mucorales* spores. This review provides insights on the structural composition of the *Mucorales* cell wall and potential enzymes involved in cell wall biosynthesis.

## *Mucorales* Cell Wall Polysaccharides

Studies dealing with the structural composition of the *Mucorales* cell wall are scarce and have mainly focused on *Mucor mucedo* and *ucor circinelloides* (formerly *Mucor rouxii*).

### Chitosan and Chitin

As for insects and crustaceans, fungi are characterized by the presence of chitin/chitosan, which participate in the rigidity of the glycan edifice of the cell wall. Chitin is a polymer of β-(1→4)-linked GlcNAc, while chitosan is composed of a polymer of β-(1 → 4)-linked GlcNAc and more than 50% GlcNH_2_ (Table 1). One of the methods used to study chitin/chitosan in *M. mucedo* was the use of nitrous acid, which discriminates chitin and chitosan. Nitrous acid only has an effect on deamination and depolymerization if the polymer carries free –NH_2_ groups. Thereby, chitosan is degraded into 2,5-anhydromannose. Following treatment with nitrous acid, the molar ratios of anhydromannose (AnMan), GlcNAc-AnMan, GlcNAc_2_-AnMan, GlcNAc_3_-AnMan, and GlcNAc were quantified as: 67:11:3:1:13 (Datema et al., 1977a,b). The proportion of chitin/chitosan may vary between spore and hyphal forms and also between species. In *Mucorales*, these proportions were estimated at 12% and approximately 40% for spores and hyphae, respectively (Campos-Takaki et al., 2014).

**Table 1:** Partial structure of cell wall polysaccharides found in *Mucorales.*

Chitin/Chitosan	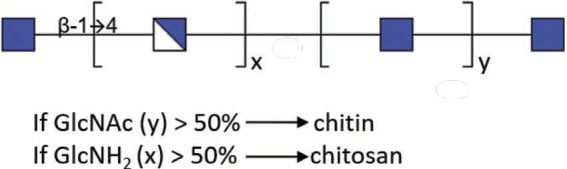
Mucoran	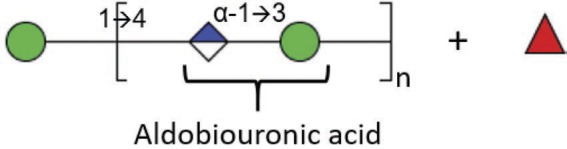
Mucoric acid	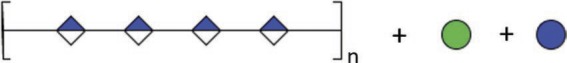
Glucan	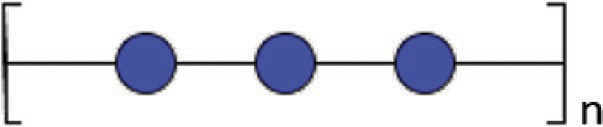
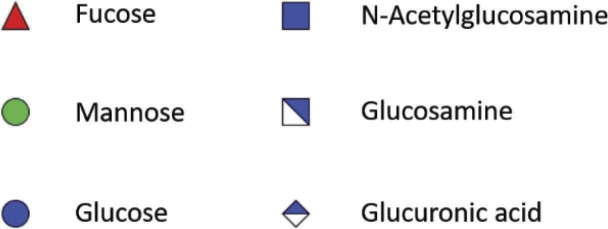	

### Glucuronic Acid

Polyuronides are polymers of glucuronic acid (GlcUA). The proportion of this polysaccharide is dependent on the stage of development of the fungus. In *M. circinelloides*, the proportion of polyuronides varies from 25% in sporangiophore walls to 12% in hyphae or yeast-like cell walls and less than 2% in spores (Bartnicki-Garcia and Reyes, 1968). In *M. mucedo*, treatment of hyphae with nitrous acid releases glycuronan that is soluble in water and linked in a non-covalent way to a polymer of glucosamine (Datema et al., 1977a,b). The proportion of glucuronic acid in these hyphal forms reaches approximately 12% (w/w). Glycuronan is composed of fucose, mannose, galactose, and glucuronic acid with a molar ratio of: 5:1:1:6. Acid hydrolysis of glycuronan leads to polymers of glucuronic acid with properties of mucoric acid previously identified by Bartnicki-Garcia and Reyes (1968). During acid hydrolysis of polyuronides, two fractions are obtained, mucoran and mucoric acid. Mucoran is an easily hydrolyzable heteropolysaccharide made of D-glucuronic acid, L-fucose, and D-mannose in a molar ratio of 5:2:3 and a small proportion of D-galactose and glucose (Bartnicki-Garcia and Lindberg, 1971). Mucoric acid is a polysaccharide resistant to acid hydrolysis composed mainly of D-glucuronic acid (Bartnicki-Garcia and Reyes, 1968).

Mucoran can be extracted from sporangiophore cell walls after hydrolysis by HCl. It can also be extracted from yeast-like cell walls by alkaline treatment with KOH and precipitation as a copper complex (Bartnicki-Garcia and Lindberg, 1971). This alkaline treatment was successfully used to partially characterize mucoran and to define its structure. Further characterization of mucoran was performed using the Hakomori method (methylation of mucoran), followed by reduction, hydrolysis, and conversion to partially methylated alditol acetates that were analyzed by gas chromatography–mass spectrometry. This analysis showed that glucuronic acid is substituted at C-4, confirmed by the presence of 2,3-di-O-methyl-D-glucose (derived from 2,3-di-O-methyl-D-glucuronic acid), and mannose is substituted at C-3, confirmed by the presence of 2,4,6-tri-O-methyl-D-mannose. Both monosaccharides were found in the aldobiouronic acid material, which is recovered after partial acid hydrolysis of mucoran. Aldobiouronic acid is a disaccharide composed of α-D-glucopyranosyluronic and acid-(1 → 3)-D-mannose. Mucoran may correspond to long chains of D-Man-(1 → [4)-α-D-GlcUA-(1 → 3)-D-Man-(1-]_n_ (Bartnicki-Garcia and Lindberg, 1971). The molecular mass of mucoran was estimated by gel filtration and varied depending on the form of the cells that the polysaccharide was derived from. Its molecular mass varied from 19.2 kDa in the mycelial form to 34.8 kDa in yeast-like cells (Dow et al., 1983a,b). In contrast to mucoran, mucoric acid has a similar molecular mass (32–33 kDa) in sporangiophores, mycelium, or yeast-like cell walls. The monosaccharide composition revealed that mucoric acid is composed of >90% glucuronic acid, 5% glucose, 2% mannose, and traces of galactose and fucose (Dow et al., 1983a,b) (Table 1).

### Glucan and Melanin

In contrast to hyphae or yeast-like cells, glucan was found to be the major component of the spore cell wall in *M. circinelloides* in association with melanin, glucosamine, mannans, and proteins. The corresponding abundance has been quantified in spore cell walls as follows: 9.5% glucosamine, 2.1% GlcNAc, 42.6% glucose, 4.8% mannose, 16.1% proteins, 10% lipids, 2.6% phosphate, and 10.3% melanin (Bartnicki-Garcia and Reyes, 1964). This study suggests that glucan synthesis may decrease during spore germination leading to modification of the cell wall glycoshield. The transition from spores to hyphal cells is probably due to *de novo* synthesis of a cell wall underneath the spore wall during germination. Despite progress in our understanding of the *Candida* and *Aspergillus* cell wall structure, little is known about *Mucorales* cell wall remodeling in different growth conditions and during tissue invasion. An elegant study demonstrated the presence of glucan in *M. ramannianus* spores (Jones et al., 1968) using β-glucanase obtained from culture fluid of *Streptomyces* spp. By microscopy, the authors showed that the spore cell wall is composed of two layers: an outer electron-dense layer and an inner thicker layer composed of microfibrils containing glucans, cleavable by lytic enzymes found in culture fluid of *Streptomyces* spp. A more recent study showed the presence of β-glucan on *Rhizopus oryzae* by confocal microscopy and its role in the production of IL-23 and the triggering of T_H_-17 responses in dendritic cells *via* dectin-1 (Chamilos et al., 2010).

## *Mucorales* Extracellular Polysaccharides

*Mucorales* also secrete extracellular polysaccharides (EPS), which have been studied in several species of *Rhizopus* and *Mucor*. The structures and molecular mass of these EPS depend on the species from which they were isolated. These polysaccharides are essentially composed of β1,4-linked glucuronic acid. These EPS are composed of mannose varying from 8 to 30%; galactose from 4 to 13%; fucose from 9 to 25%; glucose from 0 to 30%; and glucuronic acid from 32 to 55% (de Ruiter et al., 1991, 1992). One study showed that mannose residues could be 2-O-methylated (de Ruiter et al., 1994). 2-O-methyl-D-mannose represents 1–2% of the two mannan fractions extracted from EPS of *M. racemosus*. Mannose residues are linked through α1,2 linkages, while 2-O-methyl-D-mannose residues are found at the non-reducing end.

## Enzymes Involved in Cell Wall Biosynthesis

### Endohydrolases

Enzymes have been implicated in biosynthesis of the fungal cell wall, notably during hyphal extension. There is a balance between synthesis and lysis of cell wall polysaccharides at the hyphal apex. Culture filtrates of *M. circinelloides* have been analyzed by gel filtration and showed the presence of uronides with several degrees of polymerization as tri-, penta-, or hexasaccharides. Analysis of these oligosaccharides showed glucuronic acid as the major part with traces of mannose, galactose, and fucose. Experiments with cycloheximide on *M. circinelloides* inhibited hyphal extension, but uronide production was unaffected suggesting a role of endohydrolases in cell wall remodeling (Dow and Villa, 1980).

### Glucuronosyl Transferase

Experiments conducted by Dow et al. (1983a,b) showed glucuronosyl transferase activity in membrane fractions from yeast-like and mycelial forms of *M. circinelloides*. This enzyme is implicated in polyuronide biosynthesis and is involved in the transfer of D-GlcUA from uridine-5′-diphosphoglucuronic acid (UDP-GlcUA) to endogenous acceptors. The acceptors are glycoproteins and polyuronides, such as mucoric acid and mucoran, found in the cell wall. The enzymes are characterized by a pH optimum between 7.0 and 8.0 for mycelial extracts, but enzymes found in yeast extracts have a lower optimum pH varying from 6.5 to 7.0. Mn^2+^ ions are required for maximal activity of enzymes from both sources (Flores-Carreón et al., 1985).

### Fucosyl Transferase

As fucose is found in the zygomycete cell wall, there are probably some enzymes involved in the transfer of L-fucose from GDP-fucose to polysaccharides. Fucosyl transferase activity has been partially characterized and detected in membrane fractions from *M. circinelloides*. This fucosyl transferase has optimal activity at pH 6.5 and at a temperature between 22 and 28°C. This enzyme also requires the presence of metal divalent cations such as Mn^2+^, Mg^2+^, Co^2+^, Zn^2+^, Fe^2+^, and Ca^2+^. It has been shown that mucoric acid was not only an acceptor of fucosyl residues but also an activator of the enzyme (Camacho-Aguero et al., 1990).

### Chitin Synthase and Deacetylase

Chitin synthesis is catalyzed by the transfer of GlcNAc from UDP-GlcNAc to chitin by chitin synthase. Chitosan is produced by deacetylation of chitin by chitin deacetylase. A chitin deacetylase has been partially purified and characterized from *M. circinelloides*. It was found in the supernatant of disrupted mycelia of *M. circinelloides* centrifuged at 20000 *g* and in culture media. This enzyme seems to be cytoplasmic and is released into culture media (Araki and Ito, 1975). Chitosan synthesis is not only due to chitin deacetylase activity; this enzyme works in tandem with a chitin synthase (Davis and Bartnicki-Garcia, 1984a,b). In this study, the authors showed that chitin is a precursor of chitosan. They also showed that incubation of both enzymes with UDP-GlcNAc led to the conversion of 10–15% of the substrate to chitosan within a 30-min period. The reduction in the amount of chitin was correlated with an increase in chitosan. This chitin deacetylase has an apparent molecular mass varying from 75 to 80 kDa. It is a highly mannosylated glycoprotein and the minimum substrate required for its activity is a chitotetraose. The enzyme is inhibited by carboxylic acids, particularly acetic acid (Kafetzopoulos et al., 1993).

Concerning chitin synthase, a membrane-bound enzyme was purified and characterized from *Absidia glauca* (Machida and Saito, 1993). This enzyme was purified in zymogen form and was converted to its active form by trypsin. The active form had a molecular mass of 28.5 kDa, and its activity was stimulated by GlcNAc and inhibited by UDP analogues like polyoxin D. Lending et al. (1991) also isolated 16S chitin synthase particles from cell walls of *M. circinelloides*. Several polypeptides were purified, but only a polypeptide at 21 kDa displayed enzyme activity.

### Chitinases

A chitinase has been purified from the cytosol of *M. circinelloides*. This chitinase has better activity on nascent free chitin synthesized by chitin synthase than on more polymerized chitin. This digestion leads to a release of diacetylchitobiose as the main product (Lopez-Romero et al., 1978, 1982). Organized long microfibrils of chitin seem to be less accessible to chitinases. The chitinase activity found in *M. circinelloides* corresponded to two enzymes. This enzymatic activity was also dependent on the culture age (Pedraza-Reyes and Lopez-Romero, 1989). There was a peak in activity after 4 h of culture corresponding to germination (i.e., the cell wall remodeling phase). Another peak of activity was detected after 10 h during the mid-exponential growth phase. Chitinase I has a molecular mass of 30 kDa, whereas chitinase II has a molecular mass of 24 kDa.

### Chitosanases

Two endochitosanases, A and B, were purified from autolyzed cultures of *M. circinelloides*. Their molecular masses were 76 kDa and 58 kDa, respectively. These enzymes released the dimer and trimer of glucosamine, respectively, derived from chitosan (Alfonso et al., 1992).

### 1,3-β-Glucan Synthase

In fungal pathogens, such as *A. fumigatus* and *C. albicans*, glucan synthesis is well documented and involves a multi-subunit complex composed of an integral membrane protein and a regulatory subunit, encoded by members of the *FKS* and *RHO1* gene families. The presence of the *FKS* gene was also reported in *Rhizopus oryzae* (Ibrahim et al., 2005). The gene was cloned and sequenced. The deduced amino acid sequence revealed 64% conservation with other members of the *Fksp* family found in *S. cerevisiae, C. albicans, C. neoformans*, and *A. fumigatus*. Glucan synthase activity was measured by incubation of crude membranes from *R. oryzae* with UDP-glucose. Radiolabeled products were found and were susceptible to digestion with exo-1,3-β-D-glucanase. This activity was inhibited by caspofungin.

More recently, the genome sequence of *R. oryzae* strain 99–880 has been published using Sanger sequencing technology (Ma et al., 2009). A large number of glyco-enzymes potentially implicated in cell wall synthesis and remodeling have been annotated following the CAZy annotation pipeline (Battaglia et al., 2011). Notably, *R. oryzae* genome contains 21 CAZymes related to chitin cell wall modification and recycling including 34 chitin deacetylases; 43 related to chitosan including 3 chitosanases; 27 related to β1,3-glucan including 3 putative 1,3-β-D-glucan synthases; 5 related to α-1,3-glucan; and 7 related to β-1,6-glucan. However, these last groups of genes would have a role in the cell wall degradation of other fungi like Ascomycetes.

## Sialoglycoproteins and Uronic Acid-Containing Glycoproteins

Glycoproteins were found in the cell wall of *Mucorales* that could interact with host cells. Recently, an attempt was made to characterize the nature of these glycoproteins. For this purpose, lectin-binding assays were set up with spores and yeast cells from clinical isolates of the dimorphic fungus *M. polymorphosphorus*. The spores were incubated with lectins with an affinity for sialic acids, such as *Limulus polyphemus* (LPA), *Sambucus nigra* (SNA), and *Maackia amurensis* (MAA) lectins. SNA recognizes α-2,6 linked sialic acids, MAA recognizes α-2,3 linked sialic acids, and LPA recognizes sialic acids with any type of linkage. These authors demonstrated the presence of both α-2,6- and α-2,3-linked sialic acid residues in the spore cell wall. By Western blot, they also showed that the sialic acid residues were found on cell wall glycoproteins. Sialic acids seem to protect fungi against phagocytosis by human neutrophils and monocytes (Almeida et al., 2013).

Glycoproteins containing uronic acid were also detected in the cytoplasm and cell wall from *M. circinelloides.* These proteins had a molecular mass of 16.5 kDa. The calculated ratio of protein/uronic acid was 0.33, representing two-thirds of the glycan part of these proteins. It was suggested that these glycoproteins could be a potential acceptor for chain initiation during polyuronide biosynthesis (Mormeneo et al., 1995).

## Conclusion

**Figure 1:**
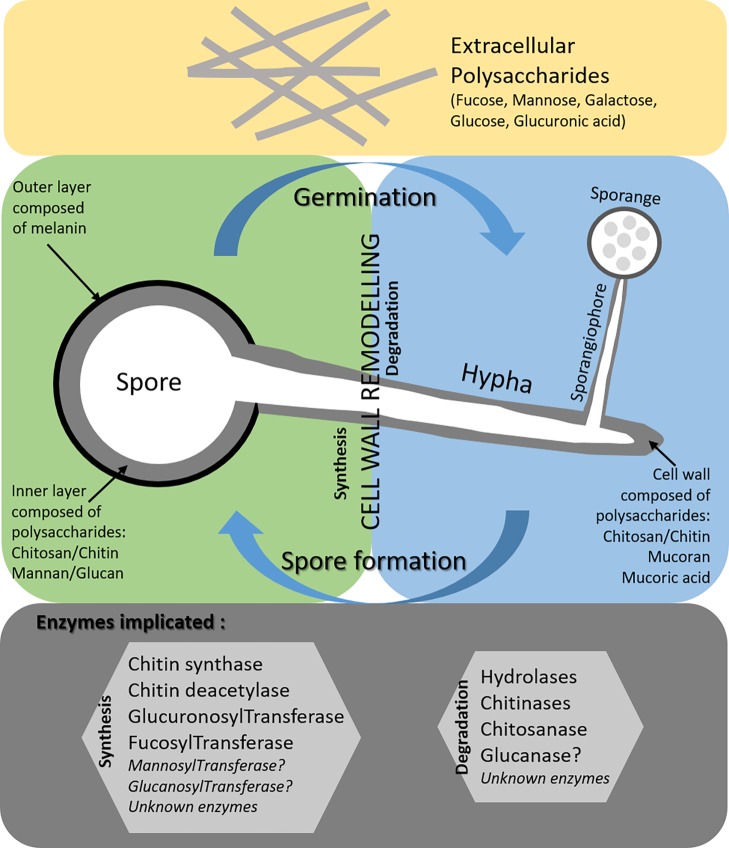
Current knowledge on the cell wall of *Mucorales* spores (green part) and hyphae (blue part) and gaps (gray part).

Compared to other fungal pathogens, the precise structure of *Mucorales* cell wall remains unknown. Some polysaccharides have been described such as mucoran, mucoric acid, chitin, and chitosan for *M. circinelloides and M. mucedo*. The proportion of these polysaccharides in other *Mucorales* spp. involved in human infections remains to be determined (i.e., *Lichtheimia corymbifera, R. arrhizus*, and *R. microsporus)*. Contrary to generally accepted ideas, the presence of β-glucan in the cell wall of *M. circinelloides* and *R. oryzae* spores was reported in 1964 and 2005, respectively. However, only two studies have described the existence of β-1,3 glucan synthase similar to that found in other fungal species belonging to the Ascomycetes (Ibrahim et al., 2005; Angebault et al., 2016). Mannose is also found in the cell wall of *Mucorales*, either in polysaccharides or in glycans. Moreover, the *ALG2* gene was reported in *R. pusillus* and was considered as a homologue of genes found in *S. cerevisiae* and encoding a glycosyltransferase playing a role in the mannosylation of Man2GlcNAc2-dolichol diphosphate (PP-Dol) and Man1GlcNAc2-PP-Dol leading to Man3 GlcNAc2-PP-Dol (Yamazaki et al., 1998). Protein O-mannosyltransferase, reported in the most common medically important pathogens (*C. albicans* and *A. fumigatus*), has never been reported in *Mucorales* spp. For *Mucorales*, there are still many gray areas concerning the enzymes involved in the biosynthesis and degradation of the cell wall, notably during the germination of spores (Figure 1). The role of Mucorales cell wall polysaccharides in triggering host immune response is not clear. Stimulation of immune response is dependent of the developmental stage of Mucorales (spores, germ tube or hyphae). Concerning the early steps of dissemination in blood vessels, it was reported that Rhizopus spores adhere to laminin and type IV collagen of the basement membranes (Bouchara et al., 1996). Moreover, germinating spores of *Rhizopus* interact with epithelial cells by a specific recognition through GRP78, a host receptor glucose-regulator protein on host membrane cells, and CotH3, a protein belonging to the spore coating protein family (Gebremariam et al., 2014). Concerning phagocytic cells, macrophages can inhibit spore germination but cannot kill them. When spores are germinating, they are more susceptible to the damage by macrophages and neutrophils (Ghuman and Voelz, 2017) probably linked to the exposure of PAMPs of the cell wall. A better knowledge of the cell wall structure in term of polysaccharides will improve our understanding of the interaction between the host and *Mucorales* pathogens and will promote a better management of human infections.

## Author Contributions

KL and BS developed the ideas and drafted the manuscript. MC, JL, and PC collected the literatures and drew the figures. KL and BS professionally edited the manuscript.

### Conflict of Interest Statement

The authors declare that the research was conducted in the absence of any commercial or financial relationships that could be construed as a potential conflict of interest.

## References

[ref1] Alfonso C. Jesús Martínez M. Reyes F. (1992). Purification and properties of two endochitosanases from *Mucor rouxii* implicated in its cell wall degradation. FEMS Microbiol. Lett. 95, 187–194. 10.1111/j.1574-6968.1992.tb05364.x

[ref2] Almeida C. A. de Camos-Takaki G. M. Portela M. B. Travassos L. R. Alviano C. S. Alviano D. S. (2013). Sialoglycoproteins in morphological distinct stages of *Mucor polymorphosphorus* and their influence on phagocytosis by human blood phagocytes. Mycopathologia 176, 183–189. 10.1007/s11046-013-9692-6, PMID: 23943428

[ref3] Angebault C. Lanternier F. Dalle F. Schrimpf C. Roupie A. L. Dupuis A.. (2016). Prospective evaluation of serum β-glucan testing in patients with probable or proven fungal diseases. Open Forum Infect. Dis. 3ofw128. 10.1093/ofid/ofw128, PMID: 27419189 PMC4942764

[ref4] Araki Y. Ito E. (1975). A pathway of chitosan formation in *Mucor rouxii*. Enzymatic deacetylation of chitin. Eur. J. Biochem. 55, 71–78. 10.1111/j.1432-1033.1975.tb02139.x, PMID: 240696

[ref5] Bartnicki-Garcia S. Reyes E. (1964). Chemistry of spore wall differentiation in *Mucor rouxii*. Arch. Biochem. Biophys. 108, 125–133. 10.1016/0003-9861(64)90363-7, PMID: 14233903

[ref6] Bartnicki-Garcia S. Reyes E. (1968). Polyuronides in the cell walls of *Mucor rouxii*. Biochim. Biophys. Acta 170, 54–62. 5721922 10.1016/0304-4165(68)90160-8

[ref7] Bartnicki-Garcia S. Lindberg B. (1971). Partial characterization of mucoran: the glucuronomannan component. Carbohydr. Res. 23, 75–85. 10.1016/s0008-6215(00)81579-7 5050614

[ref411] Battaglia E. Benoit I. van den Brink J. Wiebenga A. Coutinho P. M. Henrissat B.. (2011). Carbohydrate-active enzymes from the zygomycete fungus Rhizopus oryzae: a highly specialized approach to carbohydrate degradation depicted at genome level. BMC Genomics 12:38. 10.1186/1471-2164-12-38, PMID: 21241472 PMC3032700

[ref8] Bitar D. Lortholary O. Le Strat Y. Nicolau J. Coignard B. Tattevin P.. (2014). Population-based analysis of invasive fungal infections, France, 2001-2010. Emerg. Infect. Dis. 20, 1149–1155. 10.3201/eid2007.140087, PMID: 24960557 PMC4073874

[ref9] Bitar D. Van Cauteren D. Lanternier F. Dannaoui E. Che D. Dromer F.. (2009). Increasing incidence of zygomycosis (mucormycosis), France, 1997-2006. Emerg. Infect. Dis. 15, 1395–1401. 10.3201/eid1509.090334, PMID: 19788806 PMC2819884

[ref10] Bouchara J. P. Oumeziane N. A. Lissitzky J. C. Larcher G. Tronchin G. Chabasse D. (1996). Attachment of spores of the human pathogenic fungus *Rhizopus oryzae* to extracellular matrix components. Eur. J. Cell Biol. 70, 76–83. PMID: 8738422

[ref11] Camacho-Aguero S. Balcazar-Orozco R. Flores-Carreón A. (1990). Biosynthesis of polyuronides in *Mucor rouxii*: partial characterization of fucosyl transferase. Exp. Mycol. 14, 227–233. 10.1016/0147-5975(90)90020-T

[ref12] Campos-Takaki G. M. Dietrich S. M. C. Beakes G. W. (2014). “Cytochemistry, ultrastructure and x-ray microanalysis methods applied to cell wall characterization of Mucoralean fungi strains” in Microscopy: Advances in Scientific Research and Education. ed. Mendez-Vilas A. (Formatex), 121–127.

[ref13] Chamilos G. Ganguly D. Lande R. Gregorio J. Meller S. Goldman W. E.. (2010). Generation of IL-23 producing dendritic cells (DCs) by airborne fungi regulates fungal pathogenicity via the induction of T_H_-17 responses. PLoS One 5:e12955. 10.1371/journal.pone.0012955, PMID: 20886035 PMC2944889

[ref14] Datema R. van den Ende H. Wessels J. G. (1977a). The hyphal wall of *Mucor mucedo*. 1. Polyanionic polymers. Eur. J. Biochem. 80, 611–619. 923597 10.1111/j.1432-1033.1977.tb11918.x

[ref15] Datema R. Wessels J. G. van den Ende H. (1977b). The hyphal wall of Mucor mucedo. 2. Hexosamine-containing polymers. Eur. J. Biochem. 80, 621–626. 923598 10.1111/j.1432-1033.1977.tb11919.x

[ref16] Davis L. L. Bartnicki-Garcia S. (1984a). Chitosan synthesis by the tandem action of chitin synthetase and chitin deacetylase from *Mucor rouxii*. Biochemistry 23, 1065–1073.

[ref17] Davis L. L. Bartnicki-Garcia S. (1984b). The coordination of chitosan and chitin synthesis in *Mucor rouxii*. J. Gen. Microbiol. 130, 2095–2102. 6470680 10.1099/00221287-130-8-2095

[ref18] de Ruiter G. A. van der Lugt A. Voragen A. Rombouts F. Notermans S. (1991). High-performance size-exclusion chromatography and ELISA detection of extracellular polysaccharides from Mucorales. Carbohydr. Res. 215, 47–57. 10.1016/0008-6215(91)84006-Z

[ref19] de Ruiter G. A. Josso S. Colquhoun I. Voragen A. Rombouts F. (1992). Isolation and characterization of β(1-4)-D-glucuronans from extracellular polysaccharides of moulds belonging to Mucorales. Carbohydr. Polym. 18, 1–7. 10.1016/0144-8617(92)90181-O

[ref20] de Ruiter G. A. Van Bruggen-Van der Lugt A. W. Mischnick P. Smid P. Van Boom J. H. Notermans S. H. (1994). 2-O-methyl-D-mannose residues are immunodominant in extracellular polysaccharides of *Mucor racemosus* and related molds. J. Biol. Chem. 269, 4299–4306. 8307996

[ref21] Dow J. M. Villa V. D. (1980). Oligoglucuronide production in *Mucor rouxii*: evidence for a role for endohydrolases in hyphal extension. J. Bacteriol. 142, 939–944. PMID: 7380812 10.1128/jb.142.3.939-944.1980 PMC294120

[ref22] Dow J. M. Darnall D. W. Villa V. D. (1983a). Two distinct classes of polyuronide from the cell walls of a dimorphic fungus, *Mucor rouxii*. J. Bacteriol. 155, 1088–1093. 6885716 10.1128/jb.155.3.1088-1093.1983 PMC217802

[ref23] Dow J. M. Olona P. M. Villa V. D. (1983b). Glucuronosyl transferase from the dimorphic fungus *Mucor rouxii*. Exp. Mycol. 6, 329–334.

[ref24] Flores-Carreón A. Balcazar R. Rui’z-Herrera J. (1985). Characterization of glucuronosyl transferase from *Mucor rouxii*: requirement for polyuronide acceptors. Exp. Mycol. 9, 294–301. 10.1016/0147-5975(85)90002-7

[ref25] Gebremariam T. Liu M. Luo G. Bruno V. Phan Q. T. Waring A. J.. (2014). CotH3 mediates fungal invasion of host cells during mucormycosis. J. Clin. Invest. 124, 237–250. 10.1172/JCI71349, PMID: 24355926 PMC3871245

[ref26] Gomes M. Z. R. Lewis R. E. Kontoyiannis D. P. (2011). Mucormycosis caused by unusual mucormycetes, non-Rhizopus, −Mucor, and -Lichtheimia species. Clin. Microbiol. Rev. 24, 411–445. 10.1128/CMR.00056-10, PMID: 21482731 PMC3122490

[ref27] Gow N. A. R. Latge J. P. Munro C. A. (2017). The fungal cell wall: structure, biosynthesis, and function. Microbiol. Spectr. 5, 1–25. 10.1128/microbiolspec.FUNK-0035-2016 PMC11687499 28513415

[ref28] Ghuman H. Voelz K. (2017). Innate and adaptive immunity to Mucorales. J. Fungi 3, 48. 10.3390/jof3030048 PMC5715954 29371565

[ref29] Ibrahim A. S. Bowman J. C. Avanessian V. Brown K. Spellberg B. Edwards J. E. Jr.. (2005). Caspofungin inhibits *Rhizopus oryzae* 1,3-beta-D-glucan synthase, lowers burden in brain measured by quantitative PCR, and improves survival at low but not a high dose during murine disseminated zygomycosis. Antimicrob. Agents Chemother. 49, 721–727. 10.1128/AAC.49.2.721-727.2005, PMID: 15673756 PMC547300

[ref30] Jones D. Bacon J. S. D. Farmer V. C. Webley D. M. (1968). Lysis of cell walls of *Mucor ramannianus* Möller by a Streptomyces sp. Antonie Van Leeuwenhoek 34, 173–182. 10.1007/BF02046428, PMID: 5301324

[ref31] Kafetzopoulos D. Martinou A. Bouriotis V. (1993). Bioconversion of chitin to chitosan: purification and characterization of chitin deacetylase from *Mucor rouxii*. Proc. Natl. Acad. Sci. USA 90, 2564–2568. 8464862 10.1073/pnas.90.7.2564 PMC46135

[ref32] Latgé J. P. Calderone R. (2002). Host-microbe interactions: fungi invasive human fungal opportunistic infections. Curr. Opin. Microbiol. 5, 355–358. 10.1016/S1369-5274(02)00343-0, PMID: 12160852

[ref33] Latgé J. P. (2010). Tasting the fungal cell wall. Cell. Microbiol. 7, 863–872. 10.1111/j.1462-5822.2010.01474.x 20482553

[ref35] Lending C. R. Leal-Morales C. A. Flores-Martinez A. Bracker C. E. Bartnicki-Garcia S. (1991). Purification and characterization of 16 S chitin synthetase particles from cell walls of *Mucor rouxii*. Exp. Mycol. 15, 11–25.

[ref36] Lopez-Romero E. Ruiz-Herrera J. Bartnicki-Garcia S. (1978). Purification and properties of an inhibitory protein of chitin synthetase from *Mucor rouxii*. Biochim. Biophys. Acta 525, 338–345. 687636 10.1016/0005-2744(78)90228-0

[ref37] Lopez-Romero E. Ruiz-Herrera J. Bartnicki-Garcia S. (1982). The inhibitory protein of chitin synthetase from *Mucor rouxii* is a chitinase. Biochim. Biophys. Acta 702, 233–236. 6211193 10.1016/0167-4838(82)90507-6

[ref410] Ma L. J. Ibrahim A. S. Skory C. Grabherr M. G. Burger G. Butler M.. (2009). Genomic analysis of the basal lineage fungus Rhizopus oryzae reveals a whole-genome duplication. PLoS Genet. 5:e1000549. 10.1371/journal.pgen.1000549, PMID: 19578406 PMC2699053

[ref34] Machida S. Saito M. (1993). Purification and characterization of membrane-bound chitin synthase. J. Biol. Chem. 268, 1702–1707. PMID: 8420947

[ref38] Mormeneo S. Zazueta-Sandoval R. Flores-Carreón A. (1995). Isolation and partial characterization of uronic acid-containing glycoproteins from *Mucor rouxii*. Curr. Microbiol. 30, 237–241. 10.1007/BF00293639, PMID: 7765897

[ref39] Pedraza-Reyes M. Lopez-Romero E. (1989). Purification and some properties of two forms of chitinase from mycelial cells of *Mucor rouxii*. J. Gen. Microbiol. 135, 211–218. 10.1099/00221287-135-1-211, PMID: 2778431

[ref40] Petrikkos G. Skiada A. Lortholary O. Roilides E. Walsh T. J. Kontoyiannis D. P. (2012). Epidemiology and clinical manifestations of mucormycosis. Clin. Infect. Dis. 54(Suppl. 1), S23–S34. 10.1093/cid/cir866 22247442

[ref41] Roden M. M. Zaoutis T. E. Buchanan W. L. Knudsen T. A. Sarkisova T. A. Schaufele R. L.. (2005). Epidemiology and outcome of zygomycosis: a review of 929 reported cases. Clin. Infect. Dis. 41, 634–653. 10.1086/432579, PMID: 16080086

[ref42] Skiada A. Lass-Floerl C. Klimbo N. Ibrahim A. Roilides E. Petrikkos G. (2018). Challenges in the diagnosis and treatment of mucormycosis. Med. Mycol. 56, S93–S101. 10.1093/mmy/myx101, PMID: 29538730 PMC6251532

[ref43] Skiada A. Pagano L. Groll A. Zimmerli S. Dupont B. Lagrou K.. (2011). Zygomycosis in Europe: analysis of 230 cases accrued by the registry of the European Confederation of Medical Mycology (ECMM) Working Group on Zygomycosis between 2005 and 2007. Clin. Microbiol. Infect. 17, 1859–1867. 10.1111/j.1469-0691.2010.03456.x, PMID: 21199154

[ref44] Walsh T. J. Groll A. Hiemenz J. Fleming R. Roilides E. Anaissie E. (2004). Infections due to emerging and uncommon medically important fungal pathogens. Clin. Microbiol. Infect. 10, 48–66. 10.1111/j.1470-9465.2004.00839.x, PMID: 14748802

[ref45] Yamazaki H. Shiraishi N. Takeuchi K. Ohnishi Y. Horinouchi S. (1998). Characterization of alg2 encoding a mannosyltransferase in the zygomycete fungus *Rhizomucor pusillus*. Gene 221, 179–184. 10.1016/S0378-1119(98)00456-9, PMID: 9795208

